# Mobile real-time surveillance of Zika virus in Brazil

**DOI:** 10.1186/s13073-016-0356-2

**Published:** 2016-09-29

**Authors:** Nuno Rodrigues Faria, Ester C. Sabino, Marcio R. T. Nunes, Luiz Carlos Junior Alcantara, Nicholas J. Loman, Oliver G. Pybus

**Affiliations:** 1 Department of Zoology, University of Oxford, South Parks Road, Oxford, OX1 3PS UK; 2 Department of Infectious Diseases and Institute of Tropical Medicine, University of Sao Paulo, Sao Paulo, Brazil; 3 Center for Technological Innovation, Evandro Chagas Institute, Ministry of Health, Ananindeua, PA 67030-000 Brazil; 4 Department of Pathology, University of Texas Medical Branch, Galveston, TX 77555 USA; 5 Oswaldo Cruz Foundation (FIOCRUZ), Salvador, Bahia 40296-710 Brazil; 6 Institute of Microbiology and Infection, University of Birmingham, Birmingham, B15 2TT UK

## Abstract

The World Health Organization has declared Zika virus an international public health emergency. Knowledge of Zika virus genomic epidemiology is currently limited due to challenges in obtaining and processing samples for sequencing. The ZiBRA project is a United Kingdom–Brazil collaboration that aims to improve this situation using new sequencing technologies.

## Background to the Zika epidemic

On 1 February 2016, the World Health Organization declared a Public Health Emergency of International Concern in response to the transmission of Zika virus (ZIKV) in the Americas and beyond. Although ZIKV was first isolated as early as 1947, only 13 naturally occurring cases were identified in the six decades following its discovery, in Nigeria, Malaysia, and Indonesia [1]. The profile of the virus changed in 2007, when an outbreak of ZIKV was reported in the Yap islands, Micronesia, after which three-quarters of the local population are estimated to have been infected [1]. Between 2012 and 2014 the Asian genotype of ZIKV spread through Southeast Asia and the Western Pacific, before eventually reaching the Americas.

**Fig. 1:**
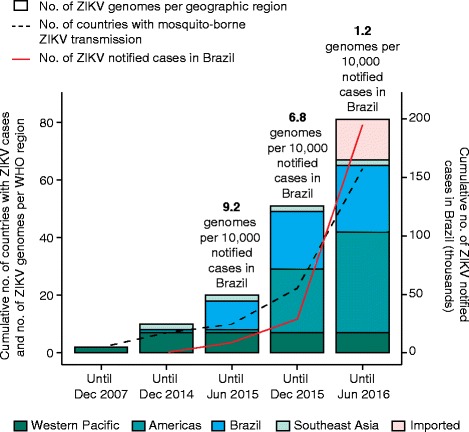
Number of whole virus genomes available for the Asian genotype of Zika virus present in the Americas and beyond. The bar plot shows the cumulative number of Zika virus (*ZIKV*) genomes from each World Health Organization (*WHO*) geographic region (see *key*). The *dashed black line* indicates the cumulative number of countries affected by the 2015–2016 ZIKV epidemic as of 14 July 2016 [5]. The *red line* indicates the cumulative number of notified cases in Brazil according to the Brazilian Ministry of Health. An estimate of the sampling proportion (number of ZIKV genomes from Brazil per 10,000 ZIKV notified cases in Brazil) is shown above each bar, from 2015 onwards. *Dec* December, *Jun* June

The first suspected cases of ZIKV infection in the Americas were detected in Brazil in early March 2015 in Natal (Rio Grande do Norte state) [2] and Camaçari (Bahia state) [3]. These two cities in the northeast of Brazil are located more than 1000 km apart. A year after its first detection, a preliminary analysis of Brazilian ZIKV genome sequences estimated that ZIKV reached the Americas between May and December 2013, most likely through a chance importation event that coincides with an increasing number of air passengers to Brazil from plausible source locations [4]. At the time of writing, the Asian genotype of ZIKV has spread to 65 countries (Fig. 1) [5] and Brazil remains the country with the highest cumulative number of cases. A growing, but still incomplete, body of evidence points towards an association between ZIKV infection and serious disease, including microcephaly in newborns and Guillain–Barré syndrome [6].

## Virus genomes in epidemiology

Viral molecular sequences can play an important role in tackling emerging epidemics and were widely used to understand the 2009 H1N1 influenza pandemic and the spread of Ebola virus in West Africa in 2013–2016 (for example, [7]). Sampled genomes enable us to quantify viral genetic diversity, reconstruct epidemic origins, estimate rates of transmission, and provide background information for vaccine development and drug design. Despite this, there is a paucity of complete genome sequences for ZIKV (Fig. 1). Until July 2016, only 23 ZIKV genomes from Brazil were publically available, yet over 190,000 suspected Zika cases have been reported to the Brazilian Ministry of Health since 2015. This equates to just 1.2 ZIKV genome sequences per 10,000 suspected infections. Consequently, we have only a fragmentary picture of the diversity of ZIKV circulating in the country. In comparison, the West African Ebola epidemic was densely sampled and sequenced; in total, researchers generated full virus genomes for more than 5 % of all known Ebola virus cases in West Africa between 2014 and 2016. Detection of ZIKV cases in Brazil is complicated by the co-circulation of Zika, dengue, and chikungunya viruses in the country [8], the clinical symptoms of which overlap with those of ZIKV, and by the fact that many Zika infections cause only mild disease and therefore go unreported. Indeed, recent estimates of the true number of ZIKV cases suggest there were 37.4 million infections during 2015 alone [9]. If true, this lowers the sampling proportion to 0.0054 sequenced genomes per 10,000 infections in Brazil during 2015.

## Dearth of Zika virus genomes

Why are so few ZIKV genomes available more than a year after the virus was discovered in the Americas? Data from the Zika Open Research Portal (https://zika.labkey.com) suggest that, in an animal model of infection, ZIKV viral load reaches a maximum only 1–2 days after virus infection. Furthermore, in humans, it seems that viral load peaks within a week of onset of clinical symptoms [1]. The early peak in viremia, combined with mild symptoms for many ZIKV infections, means that there is often little virus left for sequencing when clinical samples (usually serum) are taken from patients with suspected ZIKV infection. Consequently, it has proven comparatively difficult to obtain full ZIKV genomes directly from clinical material without enrichment (for example, http://andersen-lab.com/zika-virus-pilot). Most commonly this is undertaken by growth in cell culture, but this method is laborious and has the potential to introduce culture-specific genetic changes. An alternative approach is genome-tiling PCR, a technique used to good effect on Ebola virus but which requires labor-intensive protocol development and testing for good results.

In order to improve this situation we initiated a pilot project—the Zika in Brazil Real-time Analysis (ZiBRA) project—which aims to improve the molecular surveillance and sequencing of ZIKV in Brazil through collaboration and capacity building with a number of Brazilian research groups and institutions. ZiBRA aims to share data and results as soon as they are finished and updates are provided on the ZiBRA website (http://zibraproject.github.io). The founding ZiBRA team comprised 16 members, 14 of whom are from three Brazilian institutes: the University of Sao Paulo, FioCruz Bahia, and the Instituto Evandro Chagas. Further support has been provided by the Ministry of Health in Brazil. The project officially started in June 2016 and aims to address the following four questions.

First, what is the extent of the genetic diversity of ZIKV circulating in Brazil? This information is useful for vaccine design and for improving existing molecular diagnostic methods. Second, when and where was the virus introduced in the country? Better estimates of the dates of ZIKV introduction into each region will help epidemiologists to correctly determine the pre-ZIKV baseline level of microcephaly in each state. We will also test the hypothesis that ZIKV arrived first in Brazil, before spreading to other countries in the Americas [4]. Third, what are the trends and drivers of virus spread through Brazilian states and municipalities? We aim to build retrospective and predictive models of virus spread that will enable real-time tracking and forecasting of the spread of arthropod-borne viruses in Brazil. Fourth, are there associations between changes in the ZIKV genome and the likelihood of ZIKV complications such as microcephaly and Guillain–Barré? No such associations have yet been found, but the hypothesis is sufficiently important to warrant careful monitoring.

Between 2 and 17 June 2016, the ZiBRA project undertook fieldwork in a mobile laboratory that travelled across five federal states in the Northeast region of Brazil. This region was chosen because it contained locations with the highest numbers of notified ZIKV cases and cases of suspected microcephaly and congenital malformation. During this fieldwork we used the MinION, an innovative real-time portable genome sequencing device developed by Oxford Nanopore, which had previously been successful in characterizing the genomic diversity of Ebola virus in Guinea [7]. In Brazil, the MinION was used to perform portable whole-genome sequencing after tiling PCR.

Clinical diagnosis of suspected ZIKV cases in Brazil is performed by local clinicians, who then send patient samples (most commonly serum) to the local Central Laboratory of Public Health (LACEN), usually located in the capital city of each federal state, from where they are sent to a reference laboratory for molecular confirmation by PCR. However, during the ZiBRA fieldtrip, we found that many samples sent by the LACENs were still awaiting testing. Backlogs were caused by the unprecedented scale of the Zika public health emergency and by the fact that the laboratories must also cope with cases of suspected chikungunya and dengue virus infection, viruses that co-circulate in the same regions. Fast turnaround of molecular diagnostic results is clearly needed; some LACENs reported waiting 9 months between the date of sample collection and delivery of diagnostic results.

## Early results from the ZiBRA project

The ZiBRA team, with the help of LACEN personnel, tested 1349 samples for ZIKV RNA across Rio Grande do Norte, Paraíba, Recife, Maceió, and Bahia states, using previously described protocols [10] and the Rotor-Gene Q (Qiagen). Of these 1349 samples, approximately 14 % were sampled in 2015 and 86 % in 2016. In addition, two entomologists from Instituto Evandro Chagas, supported by personnel from local LACENs and Municipal Health Secretaries, captured more than 850 mosquitoes during the field trip from seven different species across 19 geographic locations. During the trip we trained all ZiBRA team members to perform PCR and sequencing using the Oxford Nanopore MinION platform and undertook sequencing of a set of samples. However, we found that most of the genomes generated during the fieldwork were fragmentary, often having less than 50 % coverage. This is likely due to the low amounts of virus in the samples. More recently, we developed a second version of our multiplexed tiling PCR protocol, details of which are available on the ZiBRA website (http://zibraproject.github.io). As part of our commitment to open science practices, both the protocol and materials are freely available through our website in advance of formal publication. This open analysis enables Zika research to move forward faster internationally: weeks after publishing our multiplex PCR protocol, Kristian Andersen’s laboratory at the Scripps Research Institute adopted it for sequencing of clinical samples from the United States. They rapidly modified it to support the Illumina platform and subsequently released their protocol openly for others to use (http://andersen-lab.com/miseq-protocol-zika-virus-sequencing/).

## Next steps

Complete genomic characterization of the ZIKV RNA-positive samples identified by ZiBRA will provide a framework for reconstructing the epidemic trajectories taken by ZIKV in the Americas and for tracking its spread into other geographic regions. We hope that ZiBRA will illustrate the practicality and benefits of open science and real-time data sharing during a public health emergency. Through our partnership with the Brazilian Ministry of Health, we shared all PCR results with the LACENs within 48 hours of analysis. In the future, we and a growing network of Brazilian scientists aim to extend the pilot ZiBRA project to a national scale, in order to establish a Brazilian surveillance network capable of detecting and characterizing a broad range of arboviruses in real-time.

## Abbreviations

LACEN: Central laboratory of public health
WHO: World health organization
ZiBRA: Zika in Brazil real-time analysis
ZIKV: Zika virus
